# Cross-cultural ethnobotany of the Baltis and Shinas in the Kharmang district, Trans-Himalaya India-Pakistan border

**DOI:** 10.1016/j.heliyon.2024.e28613

**Published:** 2024-03-27

**Authors:** Basharat Hussain, Zaheer Abbas, Jan Alam, Nidaa Harun, Shujaul Mulk Khan, Zeeshan Ahmad, Heesup Han, Sunghoon Yoo, António Raposo

**Affiliations:** a Department of Botany, Hazara University, Mansehra, Pakistan; b Department of Botany, Division of Science and Technology, University of Education, Lahore, Pakistan; c Department of Botany, Faculty of Life Sciences, University of Okara, Pakistan; d Department of Plant Sciences, Quaid-i-Azam University, Islamabad, Pakistan; e College of Hospitality and Tourism Management, Sejong University, 209 Neungdong-ro, Gwangjin-gu, Seoul, 05006, South Korea; f Audit Team, Hanmoo Convention Oakwood Premier, 49, Teheran-ro 87-gil, Gangnam-gu, Seoul, 06164, South Korea; g CBIOS Research Center for Biosciences and Health Technologies, Universidade Lusófona de Humanidades e Tecnologias, Campo Grande 376, 1749-024, Lisboa, Portugal

**Keywords:** Marginalized communities, Ethnobotany, Conservation, Phytocultural diversity

## Abstract

Human communities that inhabit the political borders live a transitional life, which is due to several socio-political and geo-climatic factors. The current cross-cultural ethnomedicinal study was conducted at the highly elevated Pakistan-India borders of the Western Himalayas in order to address the medicinal flora and folk knowledge of Balti and Shina communities. Ethnobotanical field surveys were conducted from May 2021 to September 2022. We used semi-structured interviews in order to collect the ethnomedicinal data. The collected data was analyzed using the relative frequency of the citations, use value, and Venn diagrams. A total of 140 interviews were conducted, which comprised of 90 (64.28%) Baltis and 50 (35.71%) Shinas. The interviews recorded 60 medicinal plant species that belong to 56 genera and 35 families. Asteraceae (5 spp.), Lamiaceae (5 spp.), and Apiaceae (4 spp.) were the families that were the most represented. These medicinally valued plants were being used for 55 health issues that are related to different body systems. *Delphinium brunonianum*, *Thymus linearis*, *Hymenolaena candollei*, *Corydalis adiantifolia*, and *Seriphidium brevifolium* were medicinal taxa with maximum RFCs and UV. The Baltis have comparatively more ethnomedicinal knowledge than the Shinas. The Baltis commonly used 22 (36.66%) species, which 34 (56.66%) were exclusive to the Baltis and 4 (6.66%) to the Shinas. Both ethnic groups collectively hold significant ethnobotanical knowledge that demands the preservation of risked folk knowledge, which is due to uncertain border situations, outmigration, and permeating allopathic drugs.

## 1 Introduction

Medicinal plants have long been some of the most used and focused on natural resources globally [1,2]. Biological diversity provides traditional medicines to 80% of the people who live in third world countries [3]. The ethnobotanical literature has recently substantially grown worldwide [4]. Ethnobotany expanded in different sub-fields or branches, such as medical, wild food, and cross-cultural ethnobotany [[5], [6], [7]]. Human culture holds ample information about their surrounding biota. The preservation of this type of information indirectly conserves the recognition, language, and culture of a particular society. Ethnobotanical knowledge is manipulated in the ethnoecological and conservation studies [8,9]. The preservation of this heritage is crucial in regards to the rapidly changing regional and global environmental scenario. The knowledge of collapsing or declining human communities due to geo-climatic threats and sociopolitical constraints makes it more urgent to document, address, and preserve. There is a significant amount of literatures found about Pakistani ethnobotany, but there are several regions that need to be explored in the north and north western uplands, which is where the maximum presentation of plant biodiversity occurs [10,11]. According to Shinwari and Qaiser [12], 12 % of the flora of Pakistan possesses medicinal effects, which mostly occurs in the mountainous regions. Gilgit-Baltistan (GB) is a typical mountainous area along the Indus River. It is also an intriguing region in terms of research activities. Abbas et al. [13] reported 413 plant species to that are used for a number of traditional therapies in Gilgit-Baltistan. Baltistan is one of the principal regions of GB, which covers several valleys, such as Rondu, Skardu, Shigar, Gultari, Khaplu, and Kharmang. The limited amount of local studies reported that the people are more vulnerable to gastro-intestinal, orthopedic, dermatological, cardiovascular, and respiratory ailments [14,15]. The hostile environmental conditions, market food trend, and sanitation discrepancy may be attributed to these health disorders. Moreover, the area is characterized by mountain ecosystems, which are inhabited by the considerable rural population. The outmigration of the natives to the town of Skardu and other cities of Pakistan is common, which is due to being a remote and border war zone with limited life necessities. These communities directly use provisioning ecosystem services, such as medicinal plants, fuel wood, fodder, and timber, for domestic needs as well as for income generation. The trend of tourism is also annually increasing. These circumstances pose threats to the existing biological resources. Fragmented exploratory botanical studies were conducted in different parts of Pakistan, such as Tormik, Shigar, Skardu, and Basho Valleys by the local botanists [11,14,[16], [17], [18]]. The Kharmang Valley is ethnobotanically unexplored. However, few species were cited in the publications regarding the flora of Pakistan. Geopolitical situations, logistic constraints, and limited facilities and accessibility hamper any type of research. Human communities that inhabit the political borders live a transitional life, which is due to several socio-political and geo-climatic factors.

The current cross-cultural ethnomedicinal study was conducted at the highly elevated Pakistan-India borders of the Western Himalayas in order to address the medicinal flora and folk knowledge of the Balti and Shina communities. The current study was conducted with three objectives, which included 1) documenting the medicinal plants of the study area, 2) determining the status and challenges for traditional knowledge retention, and 3) comparing the ethnomedicinal knowledge of the Baltis and Shina ethnic groups.

## 2 Materials and methods

### 2.1 Study area and field study

**Fig. 1:**
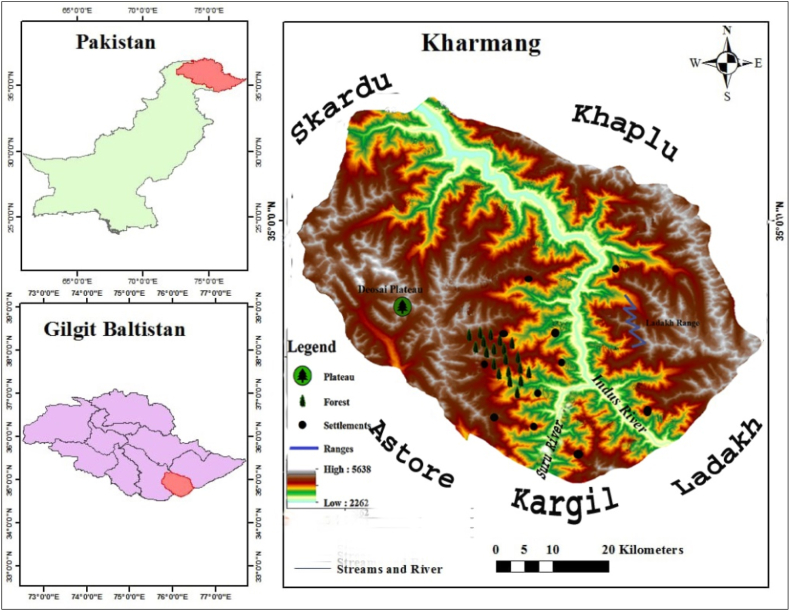
Map of the Kharmang district, Baltistan Region.

**Fig. 2:**
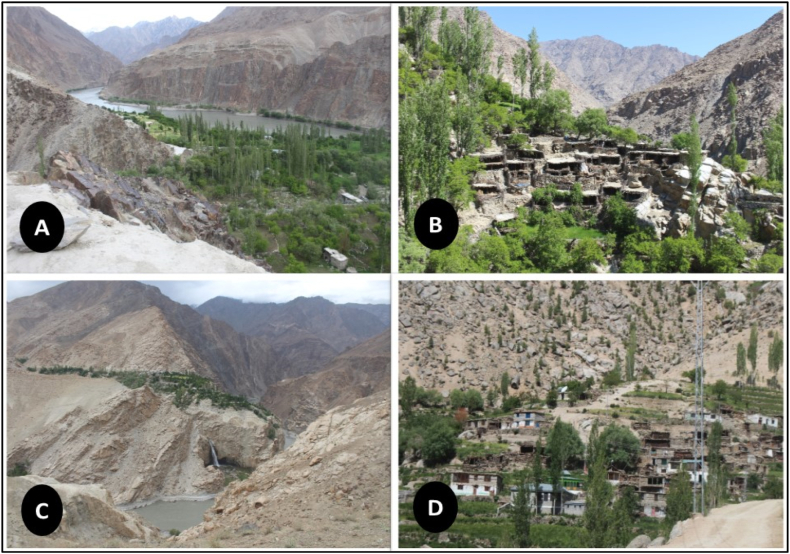
Panoramic views of some of the villages that were visited, which include (a) Tarkati, (b) Maymushthang, (c) Sheriting, and (d) Mamush.

The Kharmang Valley (34° 58′ 0″ North and 76° 14′ 0) is a newly established district of Gilgit-Baltistan (GB). It is located at one of the elevated North-eastern Indo-Pak borders. It covers an area of 7183 square kilometers, and it is in the altitudinal range of 2262–5638 m above sea level. It is geographically bounded by the Khaplu mountains from the north-east, Kargil, and Ladakh, which are Indian territories, from the south, and the Astore district from the west, which are illustrated in Fig. 1. It is the uppermost part of the Indus Valley of the Pakistani administered GB region. The area displays unique and diverse topographic features with respect to elevation, which include arid and semi-arid landscape along the Indus River and Shingo. It is rich with alluvial fans, rugged stony slopes, cliffs, gorges, snow-capped peaks, towers, and undulating foothills. The Indus River flows from the Indian territory of Kargil and confluences with the Kargil River (Suru) at the Moral Village of the Pakistani District of Kharmang. Several streams and rivulets merge with the Indus River at different localities. The climate is generally dry and hot in the summer and cold in the winter. The study area lacks a meteorological station. However, the climatic data from the only regional weather station of the Skardu town indicates the hot-arid climatic conditions in the summer. The summer temperatures are relatively high at lower altitudes, which results in the evaporation values far exceeding the precipitation, but the winter is very cold with frequent snow fall from November to March, which the average temperature remains zero degrees Celsius [19]. The mean monthly temperatures range between 6 °C and 11.5 °C with a low winter minimum of −23.2 °C. The summer is sunny and hot with a maximum of 41 °C only being reported from Skardu. The annual rainfall is between 83 and 208 mm, which is mostly received in the spring and late summer [20,21]. Vegetation is the dry temperate type, which shows ground coverage. Steppe vegetation and the occasional steppe forest groves can be found above 3500 m on the gentle slopes at the upper parts of the side valleys. Some villages are found on the gentle slopes of the riverbanks and sometimes on the rocky steeps. However, most of the settlements are found along the rivers that connect with several hanging bridges. Brolmo, Ghangani, Bilargo, and Ganokh are the highest settlements along the Shingo River, which are roughly at 2700 m above sea level. Chee-chee Thang, which is at 2800 m, Marmaq, which is at 2800 m, Danser, which is at 2700 m, and Morol, which is at 2800 m, are also the elevated villages of the Indus valley, which are shown in Fig. 2A,B,C,D. Only about 10% of the land is suitable for farming, which is partly attributable to a complex network of irrigation channels that can be up to 20 km long. Maize, wheat, barley, millet, potatoes, peas, beans, and fruit and nut trees are among the crops that are grown. Sheep, goats, cattle, and yaks are examples of livestock that provide income for the families [22]. The population of the study area is around sixty thousand that are settled in more than 250 fifty villages, which is based on the 2017 census.

### 2.2 Balti and Shina communities

The area is inhabited by two distinct minority ethnic groups, which include the *Balti* and *Shina.* The Khampa and Dardic Tibetan tribes historically migrated to Baltistan. The territory came under the rule of the Tibetan King Songtsen Gampo In the 7th century. The Bonnism and Indian Buddhism followers of the region began to embrace Tibetan Buddhism, which is due to the renewed Tibetan influence. The Islamic preachers began to come from the Arab and Persia regions to Baltistan from different mountain tracks of Kashmir in the 9th century. The people embraced Islam, and they made Baltistan an extreme extension of Islam in the mountain belt. They have been Muslims for 5 centuries, but they speak the ancient Tibeto-Burman language of Balti. It is basically one of the five Western Tibetan languages of the Sino-Indian and Pakistan mountain belts. It is spoken in more than 30 villages of the Indian Ladakh district. More than 350,000 people speak the Balti language in Baltistan. The study area shows that the Baltis are typically settled at lower elevations along the bank of the Indus River, but the Shinas are located at elevated villages except for Tarkati Village. The Shina are mostly settled at higher elevations on the side of a stream that runs down from higher altitudes. A few villages, which include Umbolung, Bilargo, Brasil Choo, Skilma, and Ghanghani, with genuine Tibetan inhabitants are also settled in the study area near the Pakistan-Indian border. The rest of the Balti accents in the lower areas are related to the Baltistan region. Shina is an Indo-Aryan language that is spoken by over 1,150,000 people in Pakistan. It is one of the major languages that is spoken in GB, which is mainly in the Gilgit region and partially in Baltistan (Mumtaz et al. [23]. The Shina people of Gilgit Baltistan migrated from Central Asia during the first half of the second millennium BC. These people are thought to have arrived in Pakistan from Central Asia via the Khyber, Pakhli above Darband, Siran, and then Kohistan. The population of the community is less than that the population of the Balti community [22,23]. The actual advent of these people in GB is unknown. However, they are believed to come from different areas of Ladakh, Kishenganga and Dras valley Jammu, and Kashmir, India, which is based on the history of the adjacent areas. Most of the anthropologists believe that the two Shina speaking tribes, which include the Shins and Yashkuns, are almost certainly from the Indo-Aryan ancestry, because they speak the Dardic language. According to the study area, they are settled in high altitude villages. They have a distinct version of Shina, which is locally called Broqskat. Brasil (3100), Ghanokh (3200), Maymush (3200), and Gherakh (3000) are Shina speaking elevated villages.

### 2.3 Ethnobotanical data collection

An ethnobotanical field survey was conducted in the upper section of the Kharmang district during 2020–2021 in the elevated villages of the India-Pakistan border area. The International Society of Ethnobiology's Code of Ethics was strictly followed during the entire study period (www.ethnobiology.net/whatwe-do/coreprograms/ise-ethics-program/code-of-ethics). Ten villages in total were visited that involved six Baltis, which included Olding, Maymush thang, Bilargo, Ganghani, Chee thang, and Danser, and four of Shinas, which included Maymush, Brasil, Ghanokh, and Tarkati. Purposive and snow boll sampling techniques were employed in order to sample the respondents [4]. Semi-structured and open-ended interviews were conducted in order to obtain the ethnobotanical data after verbal consent was received from each respondent, which is shown in Appendix Table 1. The conversations were made at different places, such as homes, agricultural fields, and village gathering places, [24,25]. The preferable languages for the interviews were Balti and Shina depending upon the respondent's affiliation and background. Urdu was sometimes used as the *lingua franca* in conversation. The participants were asked to share their knowledge about their vernacular names, plant parts that are utilized, crude drug preparation, medicinal values, and any side effects. Some participants showed fresh and dried plant materials at the time of the interviews. A list of the medicinal taxa was developed, and the plant specimens were also collected with the help of local guides. The shared information was translated into English and compiled on Excel spreadsheets. The nomenclature of the plants was based on the flora of Pakistan, the flora of Ladakh [26], the flora of Deosai [27], and the flora of China [28]. The nomenclature was then confirmed by World Flora Online (www.worldfloraonline.com). All the collected plant specimens were then properly poisoned, mounted, labeled, and stored in the Herbarium of Department of Botany, Hazara University, Mansehra, Pakistan.

**Table 1:** Social and geographic characteristics of the studied site and communities.

Ethnic Group	Villages	Elevation (meter above sea level)	Number of Households	Gender (male/female)	Age groups 20–40/40-60/above 60	Number umber of interviews (male/female)	Arrival in the area	Matrimonial system	Social subsistence
**Balti**	Kenderik	3170	34	5/4	40–60	9	Autochthonous	preferred endogamy rare exogamy	Horticulture, pastoralism
	Bilargo	2580	65	5/3	40–60	8	Autochthonous	preferred endogamy rare exogamy	Horticulture, pastoralism
	Chee chy thang	2780	59	9/8	20–40	17	Autochthonous	preferred endogamy rare exogamy	Horticulture, pastoralism
	Maimushthng	2780	86	8/5	40–60	13	Autochthonous	preferred endogamy rare exogamy	Horticulture, pastoralism
	Olding	2760	560	10/9	40–60	19	Autochthonous	preferred endogamy rare exogamy	Horticulture, pastoralism
**Shina**	Brasil	3400	130	8/5	Above 60	13	Arrived in 16 century from different parts of today's Gilgit and Chilas	preferred endogamy rare exogamy	Horticulture, pastoralism
	Maimush	3300	173	12/6	Above 60	18	Arrived in 16 century from different parts of today's Gilgit and Chilas	preferred endogamy rare exogamy	Horticulture, pastoralism
	Harghosil	3380	95	11/7	40–60	18	Arrived in 16 century from different parts of today's Gilgit and Chilas	preferred endogamy rare exogamy	Horticulture, pastoralism
	Hamzigon	2540	65	8/4	40–60	12	Arrived in 16 century from different parts of today's Gilgit and Chilas	preferred endogamy rare exogamy	Horticulture, pastoralism
	Shiriting	2770	54	7/6	40–60	13	Arrived in 16 century from different parts of today's Gilgit and Chilas	preferred endogamy rare exogamy	Horticulture, pastoralism

## 3 Data analysis

### 3.1 Relative frequency citations (RFCs)

The total number of responders that indicated a species medical usage is known as the frequency of citation (FC). The relative frequency citation (RFC) was determined using this technique in order to estimate the relevance and cultural importance of each medicinal taxon in the provided communities [29].

RFCs=FCs/N

Where FC is the number of informants who mentioned a certain plant species, and N denotes the total number of informants.

### 3.2 Use value (UV)

The use value indicates the relative importance of medicinally valued species [30].

$$\text{UV}=\Sigma \frac{\text{UI}}{N}$$

Where UI is the number of informants that reported the use of a specific plant, and N is the total number of informants that reported use.

### 3.3 Venn diagram

The Venn diagrams were created in order to highlight the potential relationships between the Balti and Shina populations in terms of using plant resources in regards to curing various ailments. The data was divided into two groups using publicly accessible software depending on the target communities, and a comparison analysis was performed using proportional Venn diagrams. The number of plant species found in the study area was comparable to plant species that were found in the previous ethnobotanical studies in other Himalayan regions, which were reported by Abbas et al. [31], Abbas et al. [32], Ambu et al. [33], and Abbas et al. [34].

## 4 Results and discussion

### 4.1 The participants’ demographical information and traditional knowledge

A total of 140 people, which included 90 (64.28%) Balti and 50 (35.71%) Shina people, participated in the interview process. They represent a wide range of professional groups, which include daily wagers, farmers, government employers, herders, hunters, small scale business owners, and traditional healers (Hakims). There were 55.7% men and 44.3% women, which is shown in Table 1. The women were mainly housewives, illiterate, and engaged in their daily domestic routines. They are reclusive and limited to household activities. They do not communicate with strangers due to limited communal lives [35,36]. We noted that the older people in this area have more traditional knowledge than the younger people, which was also reported from the surrounding regions, such as Shigar, Tormik, Ladakh, and Kargil [37,38]. The ethnic knowledge of the applications of many medicinal plants had been declining in the younger population of the research area, which was observed in other areas of the Himalayan region, which might be attributed to the younger generation's lack of interest in regards to inheriting and practicing ethnomedicinal traditions [39,40]. Furthermore, the illiterate population possessed greater ethnomedicinal knowledge, which might be attributed to the fact that educated individuals are more likely to have been exposed to the industrialized world and came to rely on contemporary as opposed to traditional therapies [41].

### 4.2 Diversity and utilization of medicinal flora

**Fig. 3:**
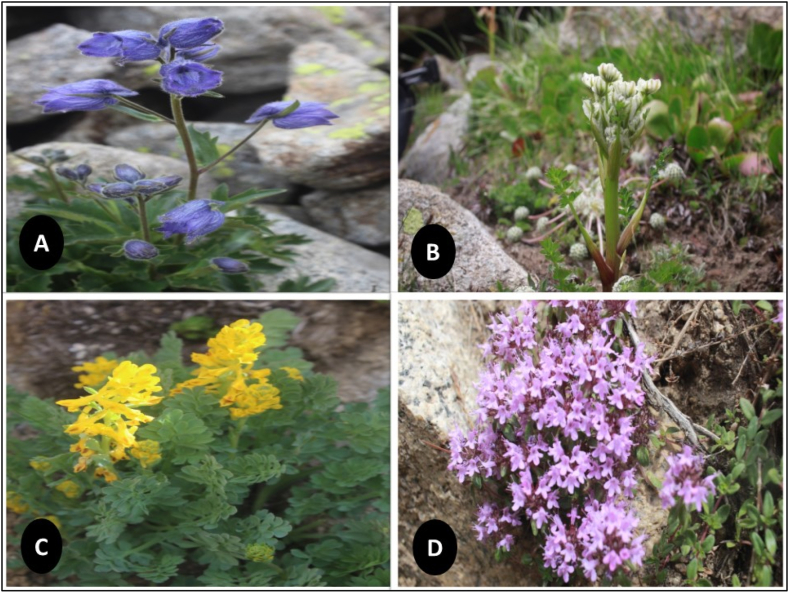
Some representative medicinal plants of the study area, which include (a) *Delphinium brunonianum,* (b) *Hymenolaena candollei,* (c) *Corydalis adiantifolia*, and (d) *Thymus linearis.*

We documented the ethnobotanical uses of 60 medicinal plants species that belong to 56 genera and 35 families. The most dominating family was Asteraceae and Labiatae among the Balti and Shina communities in the study area. They each have five different genera each, which is followed by Umbelliferae that has four genera. The other families contributed less than five species. Both groups were found to have a considerable amount of traditional knowledge of the wild medicinal plants of the Asteraceae, Lamiaceae, Umbelliferae, and Polygonaceae families. The Asteraceae family was the most prevalent, which is mainly due to their ability to adapt well to arid and dry environments due to their vast variety of ecological amplitudes. Asteraceae has been identified as a prominent family in the surrounding regions by several investigations. There was no dominancy of the genera except for Artemisia, which has three different genera that were reported. There were 43 (71%) herbs and 11 (18.33%) shrubs along with 6 (10%) tree species. The medical flora was collected from the wild with the help of a local guide. The plant growth habits were also investigated during the surveys, and three main growth habits were reported. The increased number of phytochemicals contained in leaves compared to other parts of the plant may account for their widespread usage. It is also worth noting that the accessibility and availability of a certain plant component is a major factor in its selection [42]. The herb was dominantly reported, which was followed by shrubs and trees. Leaves (28%) were identified as the most prominently used part in regards to the plant parts utilization. It was followed by whole plants (22%), flowers (14 %), roots (7%), and seeds (4%), which are illustrated in Fig. 3A,B,C,D. The area dominantly exhibits arid landscape, but it supports considerable plant taxa. More than 200 species thus far are reported to be important medicinally from Baltistan. These medicinally valued plant species are needed in order to bring in practicality by experimental cultivation, product development, and marketability. Anthropo-zoogenic threats are increasing in the regions with the passage of time. The mountain ecosystems are believed to be easily hurt by global climate change. The plants’ population would eventually decline with the associated knowledge shifting the society entirely on allopathic drugs.

**Fig. 4:**
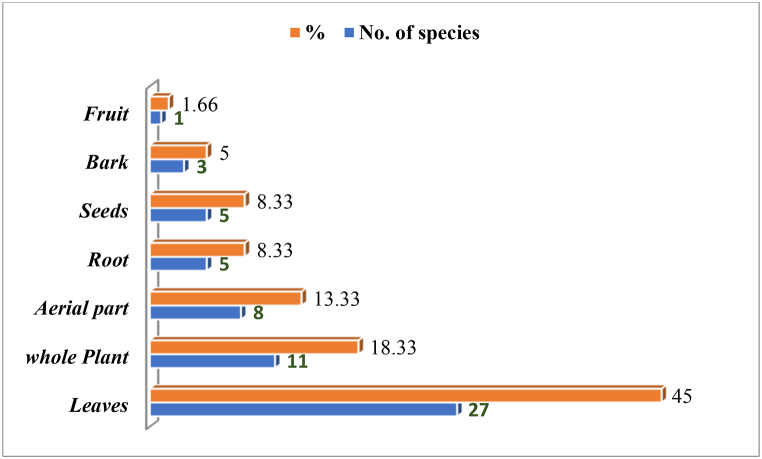
Depiction of the medicinal parts used versus the number of species.

The most traditional treatments were gastro-intestinal disorders. They were among the most treated diseases with over 15 species, which was followed by skin disorders with 7 species, respiratory tract disorders with 10 species, hepato-pancreatic with 5 species, and heart disorders with 2 species. In addition, insufficient health services in the examined areas may be one of the key probable contributors for the pathological condition, which was previously stated. Some plant species, such as *Thymus linearis*, *Delphinium brunonianum*, *Aconitum violaceum*, and *Arnebia eucroma* are also sold in the local markets, whereas *Corydalis adiantifolia*, *Hymenolaena candollei*, and *Amaranthus hybridus* are dried and preserved at home for use during the offseason. Local inhabitants, visitors, and shepherds collect a variety of plant species from higher elevations for medicinal and food uses, which include *Delphinium brunonianum*, *Pleurospermum candollei, Corydalis adiantifolia* and *Thymus linearis*. The greatest dangers to medicinal plants include grazing, trampling, and unsustainable collection. The health infrastructure in the high elevated valleys is still underdeveloped, so allopathic medications are frequently used. They erode ethnomedicinal knowledge and the traditional use of native flora, which is shown in Table 2 and Fig. 4.

**Table 2:** Medicinal flora of the upper Kharmang District Indo-Pak border, Western Himalayas, Pakistan.

Botanical Name	Family	Voucher Number	Habit	Local Name (B, Balti, S Shina)	Part(s) used	Drug formulation	Disease(s) treated	ΣUR	UV	RFC
*Acantholimon lycopodioides* (Girard) Boiss	Plumbaginaceae	HUP-14429	H	Lonzay (B/S)	Whole Plant	Ash	Heart diseases	108	0.77	0.46
*Aconitum rotundifolium* Kar. & Kir.	Ranunculaceae	HUP-14419	H	Buma (B/S)	Flower	Tea	Cough	123	0.88	0.24
*Amaranthus hybridus* L.	Amaranthaceae	HUP-14397	S	Snew; Kuno (B/S)	Leaves	Cooked	Digestive disorders	134	0.96	0.56
*Arnebia euchroma* I.M.Johnst.	Boraginaceae	HUP-14408	H	Marsee (B)	Root, stem	Root oil solution	Eye Disorders	132	0.94	0.91
*Artemesia absinthum*	Asteraceae	HUP-14418	H	Khampa (B)	whole plant	Tea	Vermifuge	123	0.88	0.25
*Artemisia rutifolia* Steph. ex Spreng.	Asteraceae	HUP-14402	S	Kar Burstae (B)	whole plant	Decoction	Digestive disorders	123	0.88	0.47
*Asparagus filicinus* Buch.-Ham. ex D.Don	Asparagaceae	HUP-14436	H	Chaaray bustee (B)	Leaves	Decoction	Digestive disorders	78	0.56	0.47
*Astragalus rhizanthus* Benth.	Fabaceae	HUP-14417	S	Bezii chuu (B); Tropo kuno (S)	The root	Raw	Teeth pain	123	0.88	0.51
*Bergenia stracheyi* (Hook.f. & Thomson) Engl.	Saxifragaceae	HUP-14411	H	Shaapull (B)	Root	Powder	Digestive ulcer	108	0.77	0.79
*Betula utilis* D.Don	Betulaceae	HUP-14421	T	Staaq Pa (B)	Bark	Raw	Bone fracture and joint dislocation	111	0.79	0.82
*Biebersteinia odora* Royle.	Biebersteiniaceae	HUP-14415	H	Chondol (B)	Flower	Tea	Vision problems	123	0.88	0.78
*Capparis spinosa* L.	Capparidaceae	HUP-14427	S	Traba (B)	Leaves	Decoction	Diabetes, joints disorders, antioxidant, anticancer and antibacterial effects.	98	0.70	0.38
*Carum carvi* L.	Apiaceae	HUP-14495	H	Dray shamdun (B); Yatrili folao (S)	Flower and Seeds	Tea	Fever and cough	12	0.09	0.05
*Chenopodium foliosum* Asch.	Chenopodiaceae	HUP-14491	H	Suyaro (S)	Aerial part	Decoction	Tumours, as antioxidant and immune stimulant.	33	0.24	0.08
*Cicer microphyllum* Royle ex Benth.	Fabaceae	HUP-14426	H	Carii (B); Khokuni (S)	Leaves and berries	Raw	Leaves and berries are edible, to cure digestive problem	111	0.79	0.37
*Cortia depressa* (D.Don) C.Norman	Apiaceae	HUP-14432	H	Sathing samdun (B)	Aerial part	Tea	Stomach pain, asthma, cholesterol, antioxidant,	98	0.70	0.78
*Corydalis adiantifolia* Hook.f. & Thomson	Fumariaceae	HUP-14393	H	Maqshang (B)	Flower	Tea	Stomach pain, tuberculosis, asthma, cold	179	1.28	0.99
*Cotoneaster nummularius* Brandis.	Rosaceae	HUP-14439	S	Sonum chespa (B)	Roots	Tea	Blood pressure	23	0.16	0.10
*Cystopteris fragilis* (L.) Bernh.	Dryopteridaceae	HUP-14493	H	Thangshing Stwa	Leaves	Decoction	Blood pressure and Cholesterol	23	0.16	0.09
*Dactylorhiza hatagirea* (D.Don) Soó	Orchidaceae	HUP-14410	H	Chu Stwa	Tuberous Root	Decoction, Dry powder	Sexual disorders, dysentery, diarrhoea, chronic fever	109	0.78	0.46
*Datura stramonium* Thunb.	Solanaceae	HUP-14405	H	Datura (B) Daturoo (S)	whole plant	Paste	Anti-inflammatory, Skin disease	56	0.40	0.49
*Delphinium brunonianum* Royle	Ranunculaceae	HUP-14582	H	Mokhooting (B)	Aerial part	Tea	Pneumonia, stomach pain, Headache, asthma, cholesterol,	232	1.66	1.00
*Delphinium cashmerianum* Royle	Ranunculaceae	HUP-14404	H	Mokhooting (B)	Aerial part	Tea	Stomach pain, Headache,	134	0.96	0.91
*Echinops cornigerus* DC./	Asteraceae	HUP-14608	H	Xoq Pilli (B); Bonser (S)	The Flower	Tea	Digestive disorder, blood pressure	45	0.32	0.13
*Elaeagnus angustifolia* Blanco	Elaeagnaceae	HUP-14428	H	Saarsing (B)	Fruits	Tea	Cough, asthma	54	0.39	0.16
*Elaeagnus rhamnoides*	Elaeagnaceae	HUP-14398	S	Karfo xoq (B)	Roots	Tea	Digestive disorders	56	0.40	0.16
*Ephedra gerardiana* Wall. ex Klotzsch & Garcke	Ephedraceae	HUP-14629	S	Sxeepat (B); Soom (S)	Leaves and berries	Powder and Paste	Asthma, Pimples on skin	128	0.91	0.91
*Euphorbia thomsoniana* Boiss.	Euphorbiaceae	HUP-14394	S	Tetri (B)	Leaves	Paste	Snake bite, highly toxic, anti-bacterial	133	0.95	0.64
*Gentiana tianschanica* Rupr.	Gentianaceae	HUP-14416	H	Ponal (B) Poonar (S)	Flower	Decoction	Eyes nourishment	123	0.88	0.69
*Heracleum pinnatum* C.B. Clarke	Apiaceae	HUP-14492	S	Spishoo (B)(S)	Seed, leaves	Raw	Inflammation, fever, abdominal cramps	32	0.23	0.06
*Hyoscyamus pusillus* L.	Solanaceae	HUP-14412	H	Lang tang (B); Lakani (S)	whole plant	Paste and decoction	Seductive, anti-bacterial	126	0.90	0.94
*Impatiens brachycentra* Kar. & Kir.	Balsaminaceae	HUP-14400	H	Sermo sing (B)	Leaves	Paste	Colour hand	102	0.73	0.32
*Iris lactea* Pall.	Iridaceae	HUP-14430	H	Trasma (B)	Leaves		Constipation	11	0.08	0.13
*Juniperus communis* L.	Cupressaceae	HUP-14424	S	Shupa (B); Chilii (S)	Branches		Skin pimples	56	0.40	0.86
*Juniperus excelsa* M.Bieb.	Cupressaceae	HUP-14395	T	Shupa (B); Chilii (S)	Seed	Paste	Urinary disorders, Ulcer	56	0.40	0.29
*Leonurus cardiaca* L.	Lamiaceae	HUP-14586	H	Kararaxchee (B)		Decoction	Antibacterial, antioxidant, anti-inflammatory and analgesic activity	45	0.32	0.14
*Lepidium latifolium* L.	Brassicaceae	HUP-14614	H	Kronbu (B)	Leaves	Decoction	Malnutrition disorders	45	0.32	0.09
*Lonicera microphylla* Willd. ex Schult.	Caprifoliaceae	HUP-14406	S	Saaid (B)	Fruits	Raw	Heart burn	98	0.70	0.43
*Mentha longifolia* L.	*Lamiaceae*	HUP-14407	H	Phololing(B)	Leaves	Decoction	Gastrointestinal disorders, vomiting, diarrhoea	129	0.92	0.24
*Myricaria germanica* (L.) Desv (L.) Desv.	Tamaricaceae	HUP-14413	S	Umboo (B)	Branches		Skin pimples	34	0.24	0.56
*Nepeta floccosa* Benth.	Lamiaceae	HUP-14585	H	Braq Samik (B)	Leaves	Powder	Cough, cold, malaria	45	0.32	0.23
*Oxyria digyna* Hill	Polygonaceae	HUP-14420	H	Squrbo (B)	Leaves	Paste	Hepatitis, Bones regenerative from fractures	122	0.87	0.91
*Peganum harmala* L.	Zygophyllaceae	HUP-14403	S	Isman (B); Mekhobi (S)	The seed	Smoke	Digestive disorders	12	0.09	0.24
*Physochlaina praealta* (Decne.) Miers	Solanaceae	HUP-14396	H	Lang tang (B); Lakani (S)	whole plant	Tea and Paste	Highly allergic and seductive, vermifuge, as anti-bacterial, anti-septic	118	0.84	0.89
*Pinus wallichiana* A.B.Jacks.	Pinaceae	HUP-14631	T	ThangShing (B)	whole plant		Skin rashes	104	0.74	0.32
*Hymenolaena candollei* DC.	Apiaceae	HUP-14630	H	Shamdun (B)	Aerial part	Tea	Stomach pain, asthma, cholesterol, antioxidant,	189	1.35	0.99
*Rheum webbianum* Royle	Polygonaceae	HUP-14399	S	Khakhol (B); Markosell (S)	Leaves	Paste	antiseptic, to cure pain in tooth	112	0.80	0.56
*Ribes orientale* Desf.	Glossulariaceae	HUP-14579	S	Askutta (B; Hargilee hummamil (S)	Whole plant	Ash	Anti-inflammation, rabies, skin infections,	135	0.96	0.96
*Rosa webbiana* Wall. ex Royle	Rosaceae	HUP-14611	S	Sia Marpo (B)	Whole plant	Decoction and tea	Inflammation of liver, Jaundices and hepatitis	132	0.94	0.94
*Rumex patientia* L.	Polygonaceae	HUP-14433	H	Shooma (B)	Leaves	Decoction and Paste	back pain, knee pain, purgative	65	0.46	0.16
*Seriphidium brevifolium* (Wall. ex DC.) Ling & Y.R. Ling	Asteraceae	HUP-14583	S	Bursay (B)	Aerial	Tea	Headache, as vermifuge, digestive disorders	170	1.21	0.98
*Sorbus tianschanica* Rupr.	Rosaceae	HUP-14434	T	Dranmo khushuu (B)	Leaves	Paste	Antiseptic, anti-bacterial	45	0.32	0.09
*Eriophyton tibeticum* (Vatke) Ryding	Lamiaceae	HUP-14401	S	Yaqq zass (B)	whole plant	Decoction	Mental disorders, fever, headache and to relieve tension.	102	0.73	0.23
*Tanacetum artemisioides* Sch.Bip. ex Hook.f	Asteraceae	HUP-14425	S	Karfoo bustee (B)	whole plant	Tea	Blood pressure and use as vermifuge	102	0.73	0.72
*Taraxacum campylodes* G.E.Haglund	Asteraceae	HUP-14409	H	Khorma (B) Lakanii (S)	Whole plant	Decoction	Blood pressure	128	0.91	0.89
*Thymus linearis* Benth.	Lamiaceae	HUP-14598	H	Tumbo Ruuk (B)Tumoro (S)	whole plant	Tea	Digestive disorders. Fever, asthma, and Cough	204	1.46	1.00
*Tribulus pentandrus* Forssk.	Zygophyllaceae	HUP-14437	H	Kukul ding (B)	Spines	Crushed	Urinary disorders	78	0.56	0.46
*Trifolium pinnatum*	Fabaceae	HUP-14414	H	Ol (B)	Flower	Tea	Cough, asthma, lungs disorders	102	0.73	0.40
*Urtica dioica* L.	Urticaceae	HUP-143431	H	Xchaaxceer (B)	Leaves	Raw	Allergy-inducing	99	0.71	0.70
*Verbascum thapsus* L.	Scrophulariaceae	HUP-14435	S	Tambaku (B); Rome Katoo (S)	Leaves and flower	Decoction	Skin infections, pulmonary problems, inflammatory diseases, asthma, spasmodic coughs	79	0.56	0.46

### 4.3 Cross-cultural comparisons and nomenclature

**Fig. 5:**
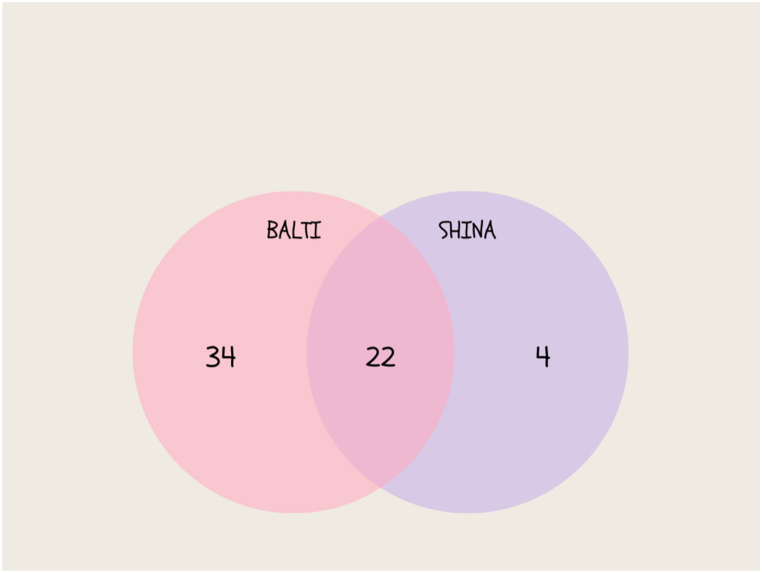
Venn diagram that illustrates the utilization of medicinal plant species among the two studied communities.

The two ethnic groups in the study area used different plant nomenclatures, but most of the uses that were reported were the same in both ethnic groups. Furthermore, some plants have similar common names, such as *Thymus linearis* Tumburuk. 20 out of the 60 therapeutic plants reported by both the Balti and Shina populations were common medicinal species, which are displayed in Fig. 5. Both groups demonstrated significant differences in the use reports of the same medicinal species when they were compared. According to a cross-cultural comparison, both study groups used more than half of the identified medicinal plants on a regular basis. The Baltis have a wealth of traditional medicinal knowledge among these two ethnic groups, which included the Balti and Shina, because they are native to the area. They have had the same usage and common names for most plant species as the Balti people. Most Balti plant nomenclature is used by the Shina ethnic group, such as *Acantholimon lycopodioides*, which has the common name *Lonzay* and *Aconitum rotundifolium*, which has the common name *Buma*. This shows that a certain cultural/linguistic group built an irreconcilable and intricate network of links with the surrounding flora. Hence, language provides a solid foundation for the preservation of TEK within a social economic zone. Furthermore, the cultural separation between the two studied groups has aided the local people in regards to articulating the local knowledge and retaining their own interpretations of natural resource utilization. Cultural isolation may have precluded the establishment of a single and standardized phytonym for each plant species, which is proven by the lack of commonality in the local names of the reported species across the two groups. Furthermore, the Baltis live at lower elevations and have a greater understanding of the medicinal properties of high mountain plant species, so they exclusively choose and employ these plants. However, the Shina community lives at higher elevations and is more reliant on the Balti community for communication and transportation to cities, and they have become more acquainted with the Balti culture. As a result, the Shina community established their local nomenclature in the area of their dominant population, such as in Indian Kashmir [[43], [44], [45]] and the lower Gilgit region of Pakistan [46,47]. It is also worth noting that the Baltis', which are former Tibetans, traditional medicinal system is heavily inspired by traditional Tibetan medicines that are passed orally and written on scripts. The disparity in the medicinal plant use reports could reflect the sociocultural limitations that have prevented traditional knowledge from being shared among ethnic groups, because they do not intermarry even though they share the same faith.

### 4.4 Quantitative analysis, novelty, and conservation

These species were utilized in the treatment of various health conditions, which include digestive, lung, skin, and hepatic disorders. They were also used for colic relief, as sedatives, and for their anti-tumor, anti-allergic, and carminative properties. Furthermore, these plants were employed in order to alleviate flatulence, gastritis, abdominal pain, coughs and colds, boils, and urinary diseases. The most often used medicinal taxa in the region are *Delphinium brunonianum*, *Thymus linearis*, *Hymenolaena candollei*, and *Corydalis adiantifolia*. Baltis and Shinas widely utilize them for pneumonia, headaches, diabetes, and stomach discomfort. *Thymus linearis* is a northern mountain species with high ecological amplitude that is harvested by the locals for domestic use as a therapeutic tea. This species can also be found in the local markets in Gilgit and Skardu [17]. This study documents the ethnomedicinal and ethnobotanical applications of 60 identified species for the first time in the region. The ethnobotanical usage was documented from nearby areas, such as Ladakh, Kargil, and Gilgit Baltistan. Most of the plants are previously documented with similar uses from the adjacent areas. Our findings demonstrate the existing state of indigenous intercultural variation in the plant resource utilization among the Balti and Shina peoples of the Kharmang. *Sorbus tianschanica*, *Tribulus pentandrus*, *Lepidium latifolium*, *Nepeta floccose*, *Cotoneaster nummularius*, *Capparis spinosa*, and *Cortia depressa* from the Baltistan region are reported for the first time. Unsustainable harvesting has put anthropogenic strain on these species. Unmanaged grazing, trampling, frequent visits, and off-road driving are all extremely harmful activities in the study region, and no meaningful attempts in regards to conserving the area's plant wealth have been implemented to date. Many species recorded from the study region are already reported from the surrounding areas of Kargil, India and Baltistan [48,49]. Furthermore, many important plants were shared by both the Baltistan and Ladakh, but the vernacular names and medical usage of these species are vastly different. The species has remained constant over time despite changes in its names and uses. This could be related to the Balti exodus from India (Ladakh) in the aftermath of the 1971 Indo-Pakistan War. Several families settled in Baltistan. Their relatives only live across the border, but they and are unable to communicate with one another due to the hostile relationship between India and Pakistan. Hence, the volume of knowledge has already been divided with the partition of the human population. The present potential factor of its decline is outmigration, which is due to the harsh lifestyle and geo-political uncertainty. Promoting home gardens, farming, the inclusion of wild plants under cultivation, and the development of organic products and marketing could augment the preservation of the existing knowledge. The proposed preservation techniques could be more productive and effective with the involvement of local botanists and the community. There may be liasoning with the national and international nutraceutical companies in order to include them in the national and international market chain.

## 5 Conclusion

This research explored the medical ethnobotany of the medicinal flora associated knowledge and practices of the two ethnic groups of the elevated border area of the Western Himalayas. They commonly and differently use a considerable number of medicinal plants. Sociocultural changes, outmigration, and transition border area circumstances are factors that cause the decline and fragmentation of the indigenous knowledge of these remote human populations. Several alpine areas are still prohibited to visit for the locals. Further extensive research can be conducted in the area if the socio-political situations permit. The existing knowledge needs to be preserved by promoting home gardens, the cultivation of wild medicinal plants, product development, and marketing. It would underpin the conservation of folk knowledge as well as increase the income generation. The present study may be a fruitful contribution for the preservation of comparative cross-cultural ethnobotany. Furthermore, it could be used in order to assess and conserve the diversity of the medicinal flora in the region.

## Funding

This is the part of M. Phil, which is the thesis of the first author, and no funding was available from any source.

## Informed consent

The research was conducted in compliance with the International Society of Ethnobiology's Code of Ethics standards. The principal author's university did not require ethics approval. Each informant gave verbal informed consent prior to every interview. The research aims and interview techniques were communicated to each informant during this session, and confidentiality was assured. Audio recording consent was also acquired. The Code of Ethics of the International Society of Ethnobiology (ISE 2008) was strictly followed.

## CRediT authorship contribution statement

**Basharat Hussain:** Writing – original draft, Visualization, Validation, Software, Methodology, Investigation, Formal analysis, Data curation. **Zaheer Abbas:** Writing – review & editing, Visualization, Validation, Supervision, Software, Resources, Project administration, Methodology, Investigation, Funding acquisition, Formal analysis, Conceptualization. **Jan Alam:** Writing – review & editing, Visualization, Validation, Supervision, Software, Resources, Project administration, Methodology, Investigation, Funding acquisition, Formal analysis, Conceptualization. **Nidaa Harun:** Writing – review & editing, Writing – original draft, Methodology, Investigation. **Shujaul Mulk Khan:** Writing – review & editing, Visualization, Validation, Supervision, Software, Resources, Project administration, Methodology, Investigation, Funding acquisition, Formal analysis, Conceptualization. **Zeeshan Ahmad:** Writing – review & editing, Writing – original draft, Methodology, Investigation. **Heesup Han:** Writing – review & editing, Resources, Funding acquisition, Data curation. **Sunghoon Yoo:** Writing – review & editing, Funding acquisition. **António Raposo:** Writing – review & editing, Resources, Funding acquisition, Data curation.

## Declaration of competing interest

The authors declare that they have no known competing financial interests or personal relationships that could have appeared to influence the work reported in this paper.
