# Supplementary material for: Cross-cultural ethnobotany of the Baltis and Shinas in the Kharmang district, Trans-Himalaya India-Pakistan border
Source: Heliyon. 2024 Mar 27;10(7):e28613. doi: 10.1016/j.heliyon.2024.e28613 (PMC10998220; doi:10.1016/j.heliyon.2024.e28613)
Supplement: Multimedia component 1 [file mmc1.docx]

Appendix Table 1: Ethnobotanical Questionnaire

**Date-** _____________**Day-** _____________________**Locality-** ____________________________________

**Respondent’s information**

**Name**- ______________­­­­­­­­­­­­­­­­­­­________ Age: _______ ___Cast**:**______________ Sub-Cast:____________________

**Education-** Illiterate**,** Primary, Middle, SSC, HSSC, Graduation, Master, Other: ____________­­______________

**Plant’s information**

**Local name-** ______________________ **Bring from -** ____________________________________________

**Carrying trend -** increasing, decreasing, no change, **Annual stock in Kilogram - ____________________ Prize per Kg -________________________________________**

**Part (s) used -** Root, stem, branches, leaves, fruit, seed, bark, bulb, rhizome, corm, whole plant, other recipe

**Drug formulation -** eaten fresh, eaten dried, decoction, infusion, paste, powder, poultice, __________________

**Disease(s) treated -** _________________________________________________________________________

**Mode of use -** Oral, topical, both oral and topical **Dosage** - Once, twice, thrice a day, as needed

**Used for-** Children, Adult, Both **Overdose side effect (s) -** ________________________________________

**Source -** Wild, cultivated Other **Use (if any) -**__________________________________________

**Remarks (If any):** __________________________________________________________________________
